# Histological evidence for secretory bioluminescence from pectoral pockets of the American Pocket Shark (*Mollisquama mississippiensis*)

**DOI:** 10.1038/s41598-020-75656-8

**Published:** 2020-10-30

**Authors:** Julien M. Claes, Jérôme Delroisse, Mark A. Grace, Michael H. Doosey, Laurent Duchatelet, Jérôme Mallefet

**Affiliations:** 1 Laboratoire de Biologie Marine, Earth and Life Institute, Université Catholique de Louvain, 1348 Louvain-la-Neuve, Belgium; 2 Biology of Marine Organisms and Biomimetics, Biosciences Institute, University of Mons, 7000 Mons, Belgium; 3 NOAA/NMFS, SEFSC/Mississippi Laboratories, 3209 Fredric St., Pascagoula, MS 39564 USA; 4 Department of Biological Sciences, University of New Orleans, 2000 Lakeshore Dr., New Orleans, Louisiana 70148 USA

**Keywords:** Marine biology, Ichthyology, Zoology, Ecology

## Abstract

The function of pocket shark pectoral pockets has puzzled scientists over decades. Here, we show that the pockets of the American Pocket Shark (*Mollisquama mississippiensis*) contain a brightly fluorescent stratified cubic epithelium enclosed in a pigmented sheath and in close contact with the basal cartilage of the pectoral fins; cells of this epithelium display a centripetal gradient in size and a centrifuge gradient in fluorescence. These results strongly support the idea that pocket shark’s pockets are exocrine holocrine glands capable of discharging a bioluminescent fluid, potentially upon a given movement of the pectoral fin. Such capability has been reported in many other marine organisms and is typically used as a close-range defensive trick. In situ observations would be required to confirm this hypothesis.

## Introduction

Pocket sharks (*Mollisquama* spp., Squaliformes) are among the most enigmatic cartilaginous fish species. Extremely rare —only one mature female and one immature male have been collected so far, from the Nazca Submarine Ridge in the southeast Pacific Ocean (*M. parini* discovered in 1984)^1^ and the central Gulf of Mexico (*M. mississippiensis*, discovered in 2010 and described in 2019)^2,3^, respectively—, these oceanic deepwater sharks also display an extraordinary combination of morphological traits including several cranial synapomorphies^4^, a strong dignathic heterodonty possibly reflecting an ectoparasitic feeding strategy, a peculiar denticle morphology, putative photogenic organ (photophore) aggregations on the ventral side and a lateral conspicuous slit above each of the pectoral fin bases^2^. These slits form the opening of the ‘pockets’ from which pocket sharks received their name. Representing a unique feature among sharks, these side pockets have puzzled scientists since their discovery, which led to many speculations about their functional significance. While Dolganov considered these glands to produce sexual pheromones^1^, more recent work suggests they might be analogous to the abdominal pouch of the Taillight Shark (*Euprotomicroides zantedeschia*), another shark species related to *Mollisquama* species, that has been observed to secrete and eject a blue bioluminescent fluid upon mechanical stimulation^2,5–7^. This pouch on the taillight shark, located immediately in front of the cloaca of both male and female specimens, shares indeed several morphological similarities with pocket shark pectoral pockets, including the presence of voluminous unscaled skinfolds raised above surrounding tissue along the slit margin and internal tightly packed villiform projections. Besides, the inner cavity of this pouch is also dark grey with a lighter bluish-grey color close to the opening. The secretory tissue of the Taillight Shark pouch is pseudostratified, made of tall columnar cells (containing small cytoplasmic granules and a large distal inclusion) separated and topped by flattened cells^5^.

In this work, we used light and fluorescence microscopy to analyze several histological sections through the right pocket of the holotype specimen of the American Pocket Shark (*M. mississippiensis*, male, 142 mm TL) as an attempt to elucidate their biological function.

## Results

**Figure 1:**
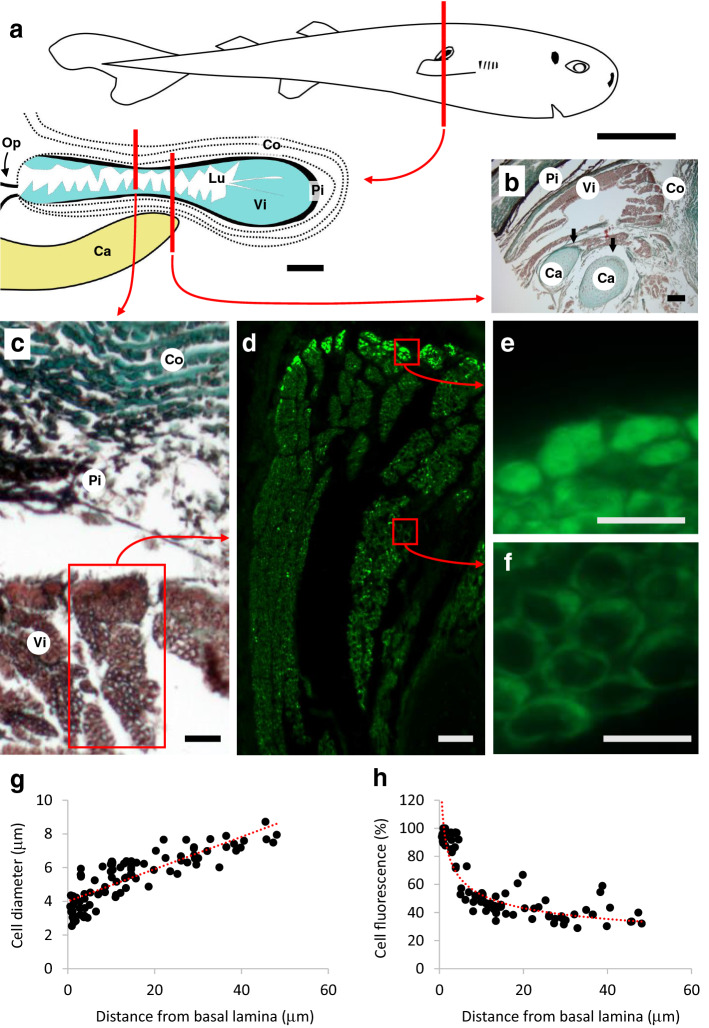
Right pocket histology of American Pocket Shark (*Mollisquama mississippiensis*). (**a**) Schematic drawing of right pocket histology in transversal view (illustration by J. M. Claes, adapted from Grace et al.^3^). *Ca* basal cartilage from the right pectoral fin, *Co, *connective tissue, *Lu *pocket lumen, *Pi* pigmented layer, *Op* pocket opening, *Vi *villiform structure. (**b**) Transversal section (Masson’s trichrome) through pocket and underlying basal cartilage from the right pectoral fin; arrows highlight the intimate connection between the cartilage elements and the villiform structures. (**c**) Transversal section (Masson’s trichrome) of pocket’s upper wall. (**d**) Green autofluorescence from a selected villiform structure in transversal section. (**e**) Diffuse bright green autofluorescence from small basal cells of the villiform structure. (**f**) Marginal weak green autofluorescence from large apical cells of the villiform structure. (**g**) Plot of villiform structure cell diameter according to the distance to basal lamina, with associated regression line (*cell diameter* = 0.10 *distance to basal lamina* + 4.00, *r*^2^ = 0.69, *n* = 100). (**h**) Plot of villiform structure cell relative fluorescence intensity according to the distance to the basal lamina, with associated regression line (*cell relative fluorescence intensity* = 101.80 (*distance to basal lamina*)^−0.29^, *r*^2^ = 0.81, *n* = 100). Indicative scale bars represent 2 cm in (**a**) (top); 1 mm in (**a**) (bottom); 10 μm in (**b–f**).

Sloping ~ 45° posteriorly to just below right pectoral fin base, the right pocket gland’s orifice had a length of ~ 4 mm (Fig. 1a) and was bordered with a series of bluish dermal folds. The interior of the gland was made of numerous darker villiform structures, which encompasses 95% of the gland volume (~ 16 mm^3^ or 0.1% of total shark volume) and its blind end was pointing anterodorsally (towards the head). No fluid was observed into the lumen of the gland but a frozen milky white fluid, potentially coming from the pocket, was observed surrounding the specimen before fixation.

Histological sections demonstrated that villiform structures reached a length of 50–150 μm and consisted in a stratified cuboidal epithelium composed of over 50 cell layers–colored in red by Masson’s trichrome (Fig. 1b,c). This epithelium was in close contact with the basal cartilage of the pectoral fin (Fig. 1b) and enclosed in a pigmented sheath surrounded by connective tissue (Fig. 1b,c). Fluorescence microscopy revealed intense green autofluorescence from villiform structures (Fig. 1d) and highlighted two cell populations: (i) small cells (2−4 μm) present close to the basal lamina and displaying a bright, homogeneously fluorescent cytoplasm (Fig. 1e) and larger cells (4−8 μm) showing only peripheral fluorescence (but with an overall larger quantity of fluorescent material) and situated between basal cells and the gland’s lumen (Fig. 1f). Due to the presence of a continuum between these two cell populations, cells composing the villiform structures displayed a centripetal gradient in size (Fig. 1g, Supplementary Spreadsheet S1) and a centrifuge gradient in fluorescence (Fig. 1h, Supplementary Spreadsheet S1).

## Discussion

Bioluminescence—e.g., the ability for an organism to produce a visible light thanks to a chemical reaction– is common in marine organisms, and especially in the permanent darkness of the deep-sea where most taxa use light to evade predators, acquire food or communicate with conspecifics^8,9^. In sharks, only two families are known to be endowed with bioluminescence capability: the Etmopteridae (Squaliformes; 53 species) and the Dalatiidae (Squaliformes; 10 species including pocket sharks and the Taillight Shark)^10,11^; phylogenetic analyses and putative photogenic organs found in *Zameus squamulosus* suggests that Somniosidae (Squaliformes) might also contain bioluminescent species^11^ but observation of light-emission displays would be needed to confirm this hypothesis. Catsharks (Scyliorhinidae, Carcharhiniformes) and carpet sharks (Orectolobidae, Orectolobiformes), on the other hand, show biofluorescence, a phenomenon distinct from bioluminescence, which results from the absorbance of the ambient down-welling light and its re-emittance at longer, lower-energy wavelengths^12^.

The pocket shark pockets are made of brightly fluorescent cells enclosed in a pigmented sheath connected to the exterior via a folded aperture and closely associated with the basal cartilage of the pectoral fin. This structure supports the hypothesis that these peculiar organs secrete and discharge a light-producing substance to the exterior in response to a specific movement of the pectoral fins (upon a given stimulation). Indeed, (1) bioluminescent compounds often display autofluorescence^8,13–16^, (2) photogenic cells (including secretory glands) from mesopelagic animals are often enclosed in light-tight pigmented sheaths to avoid unwanted bioluminescence detection^17–19^ and (3) secretory photogenic glands are sometimes associated with structures allowing fast discharge of luminous compounds^20,21^.

The ring-shaped fluorescent pattern of the cells situated at the apex of the villiform structures, which contrasts with the homogeneously fluorescent cytoplasm of basal cells, might result from a progressive accumulation of cytoplasmic inclusions in the center of the cell. Cytoplasmic inclusions have been observed in both the photocytes and secretory photogenic cells of dalatiid sharks and might be linked to luminescence competence^5,22^. The histological organisation of the villiform structures is consistent with a holocrine secretion mode, where small basal cells, accumulating fluorescent material and developing a cytoplasmic inclusion, would progressively turn into larger apical cells when approaching the lumen of the gland, finally becoming the product of the secretion^23^. Holocrine secretions are rare among secretory photophores from marine animals, which appear—based on the morphology of secretory cells and/or the examination of secretion products—to be mostly apocrine (secretion via the breaking up of the apical part of the secretory cell, resulting in the formation of extracellular membrane-bound vesicles)^21,24–27^ or merocrine (secretion via exocytosis from secretory cells)^20,28^ glands. Interestingly, the only other reported case of holocrine photogenic glands is found in tubeshoulder fishes (Searsidae), which also possess two light-producing glands opening in the close vicinity of pectoral fins^29^; such similar structural organization likely represents a case of convergent evolution. Furthermore, piscine venom glands—which are also holocrine structures lacking associated musculature—, rely on a mechanical coupling with skeleton structures (e.g., grooved spines) to discharge their secretion^30–33^, potentially representing another convergent evolution case. Surprisingly, however, the stratified cubic and putatively holocrine epithelium of the pocket shark pockets appears dramatically different from the pseudostratified columnar and putatively apocrine (based on morphology) epithelium of the pelvic photogenic pouch of the Taillight Shark^5^, suggesting an independent origin of the two organs in these closely related species. Reaching close to 10 μm in diameter, apical cells of the villiform structures reach a similar size as photocytes of juvenile dalatiid (*Squaliolus aliae*; J. M. Claes, personal observation) and etmopterid sharks (*Etmopterus spinax*)^34^. In the Slendertail Lantern Shark (*Etmopterus molleri*), photophores are known to form enlarged photogenic structure at the base of the dorsal fin^35^. Similarly, one can suppose the pocket shark pockets to be modified photophores, that merged and invaginated themselves to become a secretory gland. The phylogenetic position of *M. mississippiensis* among Dalatiidae^2,3^ supports the pocket glands to be a derived character, posterior to the presence of epidermal ventral photophores.

**Figure 2:**
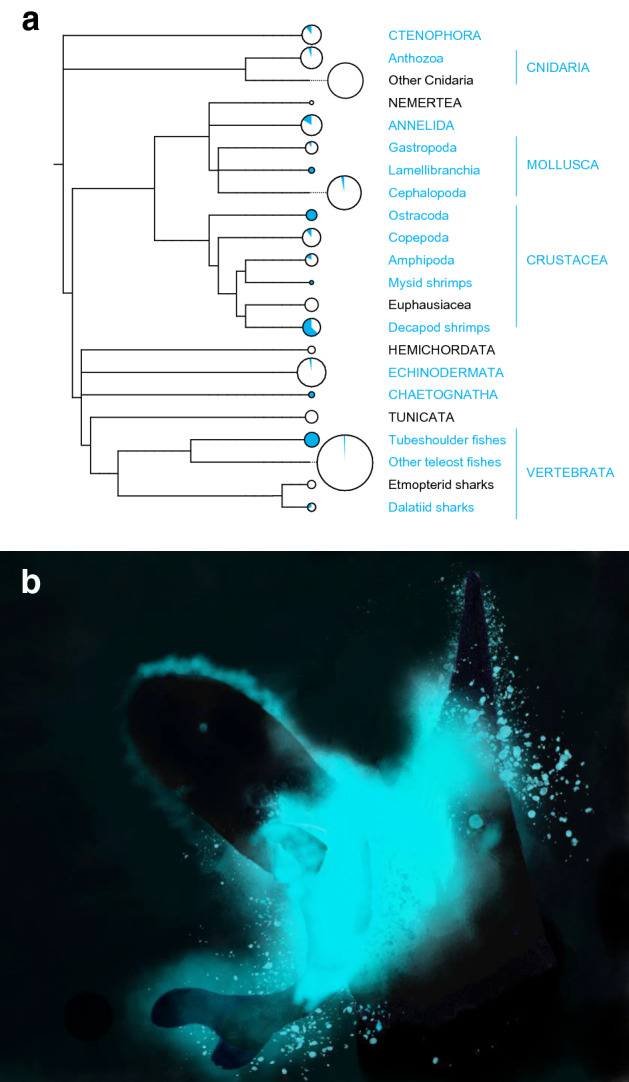
(**a**) Systematic distribution of ‘bioluminescent cloud’ emitters among 566 bioluminescent marine genera. Names in capital letters represent “phylum-level” groups or broader. Circles scale to the number of marine bioluminescent genera in a given taxonomic grouping while blue color indicates the proportion of genera with ‘bioluminescent cloud’ emitters. Blue taxonomic grouping contains at least one genus known to discharge intrinsic bioluminescent secretions outside of their body. (**b**) Artistic illustration of a putative defensive cloud-emitting behavior in an American Pocket Shark (left) following the attack of a Goblin Shark (*Mitsukurina owstoni*; right) in the darkness of the deep-sea (illustration by J. M. Claes).

Secretory luminescence is relatively uncommon among marine organisms, i.e., concerning ~ 10% of marine bioluminescent genera. However, ‘bioluminescent cloud’ emitters are found in most marine taxonomic groupings (Fig. 2a), which probably results from multiple independent origins of the cloud-emitting capability and highlights its paramount ecological significance. Although secretory luminescence is involved in intraspecific sexual courtship in ostracods^36^ and in *Odontosyllis* worms^37,38^, probably because of the energy costs associated with the production of the secretory products, the primary function of bioluminescent cloud emission appears to be last-minute short-distance defensive behaviors to facilitate escape^8^. This and the fact that the investigated specimen is not sexually mature let us hypothesize that the pocket shark pockets (and the Taillight Shark pouch) are used in similar ‘smoke screen’ predation avoidance contexts, potentially blinding approaching predators (Fig. 2b). In situ observations and additional biological material would be needed to confirm this hypothesis, even if this might require some patience given the extreme rarity of pocket sharks.

## Methods

### Histology

The holotype (TU 203676; 142.0 mm in total length and 14.6 g in total weight) of *M. mississippiensis* (which represents the unique specimen of this species) was collected in the central Gulf of Mexico on 4 February 2010 by the NOAA Ship *PISCES* (survey 7620101001, station 053) and immediately frozen in water after capture for about 3 years before being preserved in 20% formalin for 3 weeks and gradually transferred to a final solution of 70% ethanol for storage. The right pocket of this specimen was excised, cut in several subsamples and dehydrated in a graded ethanol series, with some of the subsamples fixed in Bouin’s fluid beforehand. Subsamples were then embedded in paraffin using a routine method^39^. Sections (10 µm in thickness) were cut using a Microm HM340E microtome and mounted on clean glass slides (SuperFrost Ultra Plus, Menzel GmbH & Co. KG, Germany). Sections obtained from ethanol-fixed samples were analyzed using the FITC-filter of a Zeiss Axioscope A1 microscope equipped with an AxioCam ICc3 camera (Zeiss, Oberkochen, Germany) for autofluorescence detection while sections from samples fixed in Bouin’s fluid were stained with the Masson's trichrome^39^. Digital pictures of sections were acquired using the Zeiss Zen Blue 2.5 software and finally analyzed in Image J v. 1.46 (National Institutes of Health, Bethesda, USA) to estimate the diameter (defined as square root of total 2D area) and relative fluorescent compound concentration (defined as relative fluorescence intensity) of putative light-producing cells (n = 100) constituting the pocket’s villiform structures. All experimental methods were conducted in accordance with relevant guidelines and regulations, and were approved by the NOAA/NMFS Southeast Regional Office Committee in St. Petersburg, Florida (Scientific Research Permit No. 779-1633; granting date: 9 January 2009).

### Phylogenetic tree building

A phylogenetic tree of marine bioluminescent organisms was built based on NCBI taxonomy using PhyloT^40^ and a series of representatives of each marine taxonomic grouping: *Bathyctena chuni* (Ctenophora), *Anthoptilum murrayi* (Anthozoa), *Aurelia labiata* (‘Other Cnidarias’, which encompasses the remaining bioluminescent cnidarian genera), *Hubrechtella juliae* (Nermetea)*, Odontosyllis phosphorea* (Annelida), *Doris kergelenensis* (Gastropoda), *Pholas dactylus* (Lamellibranchia), *Heteroteuthis dispar* (Cephalopoda), *Vargula tsujii* (Ostracoda), *Metridia lucens* (Copepoda), *Scina borealis* (Amphipoda), *Mysis relicta* (‘Mysid shrimps’), *Meganyctiphanes norvegica* (Euphausiacea), *Acanthephyra purpurea* (‘Decapod shrimps’), *Balanoglossus carnosus* (Hemichordata), *Ophiocomina nigra* (Echinodermata), *Caecosagitta macrocephala* (Chaetognatha), *Didemnum granulatum* (Tunicata), *Searsia koefoedi* (‘Tubeshoulder fishes’), *Porichthys notatus* (‘Other teleost fishes’, which encompasses the remaining bioluminescent teleost fish genera), *Etmopterus spinax* (‘Etmopterid sharks’) and *Isistius brasiliensis* (‘Dalatiid sharks’). The number of marine bioluminescent genera –and the proportion of these latter endowed with the capability to discharge ‘bioluminescent clouds’, e.g., intrinsic bioluminescent secretions– was determined for each taxonomic grouping by compiling information from scientific literature^8,13,24–29,36,41–55^. External bioluminescent displays resulting from autotomy (e.g., in the pelagic sea cucumber *Enypniastes eximia*^56^) were not considered as bioluminescent clouds given their non-secretory nature.

### Pocket shark’s cloud-emitting behavior illustration

The artistic illustration of the defensive cloud-emitting behavior of the American Pocket Shark’s was drawn using Adobe Photoshop Sketch (version 4.9.0). Given that both internal^57^ and external shark luminescent displays^7^ were always found to fall in the blue wavelength range (450–495 nm), we used a blue color to represent American Pocket Shark’s bioluminescent cloud. We selected the Goblin Shark (*Mitsukurina owstoni*) as the attacking predator given this species is large (up to 6 m), piscivorous and also occurs in the Gulf of Mexico’s mesopelagic zone^58^.

## Supplementary information

Supplementary Information 1.

Supplementary Information 2.

## References

[1] Dolganov VN (1984). A new shark from the family Squalidae caught on the Naska Submarine Ridge. Zool. Zh..

[2] Grace, M. A., Doosey, M. H, Bart, H. L., Naylor, G. J. First record of *Mollisquama* sp*.* (Chondrichthyes: Squaliformes: Dalatiidae) from the Gulf of Mexico, with a morphological comparison to the holotype description of *Mollisquama parini* Dolganov. *Zootaxa* **3948**,587–600 (2015). 10.11646/zootaxa.3948.3.10 25947789

[3] Grace MA, Doosey MH, Denton JS, Naylor GJ, Bart HL, Maisey JG (2019). A new Western North Atlantic Ocean kitefin shark (Squaliformes: Dalatiidae) from the Gulf of Mexico. Zootaxa.

[4] Denton JS (2018). Cranial morphology in *Mollisquama sp.* (Squaliformes; Dalatiidae) and patterns of cranial evolution in dalatiid sharks. J. Anat..

[5] Munk O, Jorgensen JM (1988). Putatively luminous tissue in the abdominal pouch of a male dalatiine shark, *Euprotomicroides zantedeschia* Hulley & Penrith, 1966. Acta Zool. (Stockh).

[6] Stehmann, M. & Krefft, G. Results of the research cruises of FRV "Walter Herwig" to South America. LXVIII. Complementary redescription of the dalatiine shark *Euprotomicroides zantedeschia* Hulley & Penrith, 1966 (Chondrichthyes, Squalidae), based on a second specimen from the western south Atlantic. *Arch. Fisch. Wiss.* **30**, 1–30 (1988).

[7] Stehmann MFW, Van Oijen M, Kamminga P (2016). Re-description of the rare taillight shark *Euprotomicroides zantedeschia* (Squaliformes, Dalatiidae), based on third and fourth record from off Chile. Cybium.

[8] Haddock SHD, Moline MA, Case JF (2009). Bioluminescence in the sea. Annu. Rev. Mar. Sci..

[9] Widder EA (2010). Bioluminescence in the ocean: origins of biological, chemical, and ecological diversity. Science.

[10] Pollerspöck, J. & Straube, N. Bibliography Database|Shark-References. www.shark-references.com (2015).

[11] Straube N, Li C, Claes JM, Corrigan S, Naylor GJ (2015). Molecular phylogeny of Squaliformes and first occurrence of bioluminescence in sharks. BMC Evol. Biol..

[12] Gruber DF, Loew ER, Deheyn DD, Akkaynak D, Gaffney JP, Smith WL, Davis MP, Stern JH, Pieribone VA, Sparks JS (2016). Biofluorescence in catsharks (Scyliorhinidae): fundamental description and relevance for elasmobranch visual ecology. Sci. Rep..

[13] Bowlby MR, Case JF (1991). Ultrastructure and neuronal control of luminous cells in the copepod *Gaussia princeps*. Biol. Bull..

[14] Robison BH, Reisenbichler KR, Hunt JC, Haddock SH (2003). Light production by the arm tips of the deep-sea cephalopod *Vampyroteuthis infernalis*. Biol. Bull..

[15] Haddock SHD, Dunn CW, Pugh PR, Schnitzler CE (2005). Bioluminescent and red-fluorescent lures in a deep-sea siphonophore. Science.

[16] Claes JM, Krönström J, Holmgren S, Mallefet J (2010). Nitric oxide in the control of luminescence from lantern shark (*Etmopterus spinax*) photophores. J. Exp. Biol..

[17] Denton EJ, Herring PJ, Widder EA, Latz MF, Case JF (1985). The roles of filters in the photophores of oceanic animals and their relation to vision in the oceanic environment. Proc. R. Soc. B.

[18] Herring, P. J. Depth distribution of the carotenoid pigments and lipids of some oceanic animals. 2. Decapod crustaceans. *J. Mar. Biol. Assoc. UK* **53**, 539–562 (1973).

[19] Herring PJ (2000). Bioluminescent signals and the role of reflectors. J. Opt. A Pure Appl. Op..

[20] Anctil M (1979). The epithelial luminescent system of *Chaetopterus variopedatus*. Can. J. Zool..

[21] Huvard AL (1993). Ultrastructure of the light organ and immunocytochemical localization of luciferase in luminescent marine ostracods (Crustacea: Ostracoda: Cypridinidae). J. Morphol..

[22] Hubbs CL, Iwai T, Matsubara K (1967). External and internal characters, horizontal and vertical distribution, luminescence, and food of the dwarf pelagic shark *Euprotomicrurus bispinatus*. Bull. Scripps Inst. Ocenogr..

[23] Schorno, S. Biogenesis of Hagfish Slime: Timing and Process of Slime Gland Refilling in Hagfishes (*Eptatretus stoutii* and *Myxine glutinosa*). (Doctoral dissertation, University of Guelph, USA, 2018).

[24] Clarke GL, Conover RJ, David CN, Nicol JAC (1962). Comparative studies of luminescence in copepods and other pelagic marine animals. J. Mar. Biol. Assoc. UK.

[25] Dilly PN, Herring PJ (1978). The light organ and ink sac of *Heteroteuthis dispar* (Mollusca: Cephalopoda). J. Zool..

[26] Bowlby, M. R., Widder, E. A. & Case, J. F. Disparate forms of bioluminescence from the amphipods *Cyphocaris faurei*, *Scina crassicornis* and *S. borealis*. *Mar. Biol.* **108**, 247−253 (1991).

[27] Gosliner TM, Vallès Y (2006). Shedding light onto the genera (Mollusca: Nudibranchia) *Kaloplocamus* and *Plocamopherus* with description of new species belonging to these unique bioluminescent dorids. Veliger.

[28] Nicol JAC (1960). Histology of the light organs of *Pholas dactylus* (Lamellibranchia). J. Mar. Biol. Assoc. UK.

[29] Nicol JAC (1958). Observations on luminescence in pelagic animals. J. Mar. Biol. Assoc. UK.

[30] Sivan G (2009). Fish venom: pharmacological features and biological significance. Fish Fish..

[31] Ziegman, R. & Alewood, P. Bioactive components in fish venoms. *Toxins* **7**, 1497–1531 (2015). 10.3390/toxins7051497 PMC4448160 25941767

[32] Borges MH, Andrich F, Lemos PH, Soares TG, Menezes TN, Campos FV, Neves LX, Castro-Borges W, Figueiredo SG (2018). Combined proteomic and functional analysis reveals rich sources of protein diversity in skin mucus and venom from the *Scorpaena plumieri* fish. J. Proteom..

[33] Gorman LM, Judge SJ, Fezai M, Jemaà M, Harris JB, Caldwell GS (2020). The venoms of the lesser (*Echiichthys vipera*) and greater (*Trachinus draco*) weever fish—a review. Toxicon.

[34] Duchatelet L, Claes JM, Mallefet J (2019). Embryonic expression of encephalopsin supports bioluminescence perception in lanternshark photophores. Mar. Biol..

[35] Duchatelet L, Pinte N, Tomita T, Sato K, Mallefet J (2019). Etmopteridae bioluminescence: dorsal pattern specificity and aposematic use. Zool. Lett..

[36] Morin JG (2019). Luminaries of the reef: The history of luminescent ostracods and their courtship displays in the Caribbean. J. Crust. Biol..

[37] Galloway TW, Welch PS (1911). Studies on a phosphorescent bermudian annelid, *Odontosyllis enopla* Verill. Trans. Am. Microsc. Soc..

[38] Markert RE, Markert BJ, Vertrees NJ (1961). Lunar periodicity in spawning and luminescence in *Odontosyllis**enopla*. Ecology.

[39] Gabe M (1968). Techniques histologiques.

[40] Letunic, I. PhyloT. https://phlot.biobyte.de (2015).

[41] Harvey EN (1917). Studies on bioluminescence: VI. Light production by a Japanese Pennatulid, *Cavernularia haberi*. Am. J. Physiol..

[42] Haneda Y (1939). Luminosity in *Rocellaria grandis* (Deshayes) (Lamellibranchia). Kagaku Nanyo.

[43] Herring PJ (1976). Bioluminescence in decapod crustacea. J. Mar. Biol. Assoc. UK.

[44] Herring PJ (1981). Studies on bioluminescent marine amphipods. J. Mar. Biol. Assoc. UK.

[45] Herring PJ (1985). Bioluminescence in the Crustacea. J. Crust. Biol..

[46] Herring PJ (1987). Systematic distribution of bioluminescence in living organisms. J. Biol. Chem..

[47] Herring PJ (1988). Copepod luminescence. Hydrobiologia.

[48] Haddock SH, Case JF (1999). Bioluminescence spectra of shallow and deep-sea gelatinous zooplankton: ctenophores, medusae and siphonophores. Mar. Biol..

[49] Deheyn DD, Latz MI (2009). Internal and secreted bioluminescence of the marine polychaete *Odontosyllis phosphorea* (Syllidae). Invert. Biol..

[50] Thuesen EV, Goetz FE, Haddock SH (2010). Bioluminescent organs of two deep-sea arrow worms, *Eukrohnia fowleri* and *Caecosagitta macrocephala*, with further observations on bioluminescence in chaetognaths. Biol. Bull..

[51] Jones A, Mallefet J (2012). Study of the luminescence in the black brittle-star *Ophiocomina nigra*: toward a new pattern of light emission in ophiuroids. Zoosymposia.

[52] Gouveneaux, A. Bioluminescence of Tomopteridae species (Annelida): multidisciplinary approach. (Doctoral dissertation, Centre National de la Recherche Scientifique, Université catholique de Louvain, Belgium, 2016).

[53] Paitio, J., Oba, Y. & Meyer-Rochow, V. B. Bioluminescent fishes and their eyes. In *Luminescence—An Outlook on the Phenomena and Their Applications.* (Thirumalai, J., Ed.) (Intech, London, 2016).

[54] Verdes A, Gruber DF (2017). Glowing worms: Biological, chemical, and functional diversity of bioluminescent annelids. Integr. Comp. Biol..

[55] Poulsen JY (2019). New observations and ontogenetic transformation of photogenic tissues in the tubeshoulder *Sagamichthys schnakenbecki* (Platytroctidae, Alepocephaliformes). J. Fish Biol..

[56] Robison BH (1992). Bioluminescence in the benthopelagic holothurian *Enypniastes eximia*. J. Mar. Biol. Assoc. UK.

[57] Claes JM, Nilsson DE, Straube N, Collin SP, Mallefet J (2014). Iso-luminance counterillumination drove bioluminescent shark radiation. Sci. Rep..

[58] Parsons GR, Ingram GW, Havard R (2002). First record of the goblin shark *Mitsukurina owstoni*, Jordan (Family Mitsukurinidae) in the Gulf of Mexico. Southeast. Nat..

