# Supplementary material for: Histological evidence for secretory bioluminescence from pectoral pockets of the American Pocket Shark (Mollisquama mississippiensis)
Source: Sci Rep. 2020 Oct 30;10:18762. doi: 10.1038/s41598-020-75656-8 (PMC7599239; doi:10.1038/s41598-020-75656-8)
Supplement: Supplementary file 2 — Supplementary Information 2. [file 41598_2020_75656_MOESM2_ESM.docx]

Supplementary Information for

**Histological evidence for secretory bioluminescence from pectoral pockets of the American Pocket Shark (*Mollisquama mississippiensis*)**

Julien M. Claes, Jérôme Delroisse, Mark A. Grace, Michael H. Doosey, Laurent Duchatelet, Jérôme Mallefet

Corresponding author: Julien M. Claes

E-mail: [julien.claes@gmail.com](mailto:julien.claes@gmail.com)

Supplementary Spreadsheet 1 is separately provided as an .xlsx file
